# Mdm20 Stimulates PolyQ Aggregation via Inhibiting Autophagy Through Akt-Ser473 Phosphorylation

**DOI:** 10.1371/journal.pone.0082523

**Published:** 2013-12-16

**Authors:** Kunihiko Yasuda, Kyoji Ohyama, Kazuko Onga, Akira Kakizuka, Nozomu Mori

**Affiliations:** 1 From the Department of Anatomy and Neurobiology, Nagasaki University School of Medicine, Nagasaki, Japan; 2 Laboratory of Functional Biology, Kyoto University Graduate School of Biostudies, Kyoto, Japan; Consejo Superior de Investigaciones Cientificas, Spain

## Abstract

Mdm20 is an auxiliary subunit of the NatB complex, which includes Nat5, the catalytic subunit for protein N-terminal acetylation. The NatB complex catalyzes N-acetylation during *de novo* protein synthesis initiation; however, recent evidence from yeast suggests that NatB also affects post-translational modification of tropomyosin, which is involved in intracellular sorting of aggregated proteins. We hypothesized that an acetylation complex such as NatB may contribute to protein clearance and/or proteostasis in mammalian cells. Using a poly glutamine (polyQ) aggregation system, we examined whether the NatB complex or its components affect protein aggregation in rat primary cultured hippocampal neurons and HEK293 cells. The number of polyQ aggregates increased in Mdm20 over-expressing (OE) cells, but not in Nat5-OE cells. Conversely, in Mdm20 knockdown (KD) cells, but not in Nat5-KD cells, polyQ aggregation was significantly reduced. Although Mdm20 directly associates with Nat5, the overall cellular localization of the two proteins was slightly distinct, and Mdm20 apparently co-localized with the polyQ aggregates. Furthermore, in Mdm20-KD cells, a punctate appearance of LC3 was evident, suggesting the induction of autophagy. Consistent with this notion, phosphorylation of Akt, most notably at Ser473, was greatly reduced in Mdm20-KD cells. These results demonstrate that Mdm20, the so-called auxiliary subunit of the translation-coupled protein N-acetylation complex, contributes to protein clearance and/or aggregate formation by affecting the phosphorylation level of Akt indepenently from the function of Nat5.

## Introduction

Protein modifications, including phosphorylation, acetylation, methylation, ubiquitination, and SUMOylation, are important for maintaining cellular homeostasis, and disruption of protein modifications can lead to cellular dysfunction during aging and the resulting age-related disorders [1-3]. Among the post-translational modifications, protein acetylation controlled by histone acetyltransferases (HATs) and histone deacetylases (HDACs) participates in transcriptional regulation through controlling the acetylation levels of histone or various transcriptional factors in the nucleus. However, in the cytoplasm, the functional significance of protein acetylation are not well understood except for α-tubulin, which is well known to stabilize microtubule networks [4]. HDAC6 and Sirt2, class II and III HDACs, respectively, deacetylate α-tubulin in the cytoplasm [5,6]. Interestingly, HDAC6 interacts with ubiquitinated proteins and regulates protein degradation using either the ubiquitin-proteasome system (UPS) [7-9] or ubiquitin-selective quality control-autophagy [10]. HDAC6 rescues neurodegeneration and closely links between autophagy and the UPS [11]. SIRT2 is also well studied in the relationship with both brain physiology (physiological brain aging) and aging-related neurodegenerative diseases (Alzheimer disease, poly glutamine (polyQ) disease, Parkinson disease) [12,13]. These findings clearly indicate that acetylation regulates, at least in part, protein clearance to maintain cellular homeostasis during the brain ageing. While recent studies showed that HDAC6 and Sirt2 have no effects on the progression of Huntington’s disease using the gene dificient mice [14,15], the relationship of the deacetylases, i.e. HDAC6 and Sirt2 with both acetylation and protein aggreagtes clearance is not brought out and the full repertoire of acetylated proteins and acetylase enzymes is mostly unknown.

 Recently, proteomic analyses of acetylated proteins have identified a number of cytoplasmic proteins that include cytoskeletal proteins, molecular chaperones, and ribosomal proteins [16-18]. These data suggest that acetylation occurs in the cytoplasm much more frequently than previously being thought. Protein acetylation occurs in two ways in internal lysine residues and at the N-terminus, i.e., Lys (K)-acetylation and N-acetylation, respectively. Approximately 80-90% of cytosolic proteins in mammals are acetylated at the N-terminal end during *de novo* protein synthesis by N-acetyltransferases (Nat) [16,19]. All the Nat complexes consist of catalytic and auxiliary subunits and are classified into five families (NatA, NatB, NatC, NatD and NatE). In humans, NatA, NatB and NatC have been identified and reported to function in translation initiation as well as other events in the cell. NatD is a specific N-terminal acetyltransferase of histone H4 and H2A [20].

The human NatA complex is composed of Ard1/Naa10 and Nat1/Naa15, which comprise the catalytic and auxiliary subunits, respectively, and catalyze co-translational acetylation of nascent polypeptides. NatA recognizes the methionine-cleaved nascent polypeptide [21,22]. On the other hand, NatA interacts with hypoxia inducible factor 1α (HIF-1α) in a manner that is independent of N-acetylation [23-26]. Secretion of amyloid precursor protein, APP, which is related to Alzheimer disease, is suppressed by interaction with the NatA complex [27]. In the case of NatB, Nat5/hNat3/Naa20 and Mdm20/Naa25 form the catalytic and auxiliary subunits, respectively. The NatB substrates have Met-Glu, Met-Asp, Met-Asn, and Met-Met sequences at their N-termini [21,22] As tropomyosin and actin have been identified as substrates of NatB in yeast, NatB is thought to regulate actin filament assembly [28-30]. In addition, knockdown of the hNatB complex inhibits cell growth and proliferation and disturbs cell cycle progression by regulating the p21 expression level [31]. Stress-induced Tfs1, which is an inhibitor of protease carboxypeptidase Y (CPY), is also a NatB substrate, and it regulates the protein kinase A pathway through N-acetyl modification [32]. Interestingly, Mdm20 is highly expressed in brain and has a possibility as an one of key molecule of neurogenesis [33].

In budding yeast, the retrograde transport of protein aggregates requires deacetyaltion of chaperonin-containing TCP1 (CCT), the yeast ortholog of Heat Shock Protein 60 (HSP60), by Sir2 (NAD-dependent deacetyltransferase) and interaction with HSP104 and tropomyosin-dependent actin cables to retain damaged and aggregated proteins in the mother cells [34]. HSPs are well known as molecular chaperones, contribute to the maintenance of protein structure. They are required for protein homeostasis from protein synthesis to protein degradation and prevent misfolding and protein aggregation [35-37]. As aforementioned, the NatB complex is required for actin-tropomyosin remodeling, and, therefore, it seems that N-acetylation *per se* or related processes may contribute to regulate protein clearance and/or aggregation control. Since Mdm20, the auxiliary subunit of NatB, is somehow highly abundant in the nervous system, we sought to explore whether Mdm20 indeed participates in protein aggregation and/or clearance control in particular in neurons. 

## Results

### Mdm20 stimulates polyQ aggregate formation

**Figure 1:**
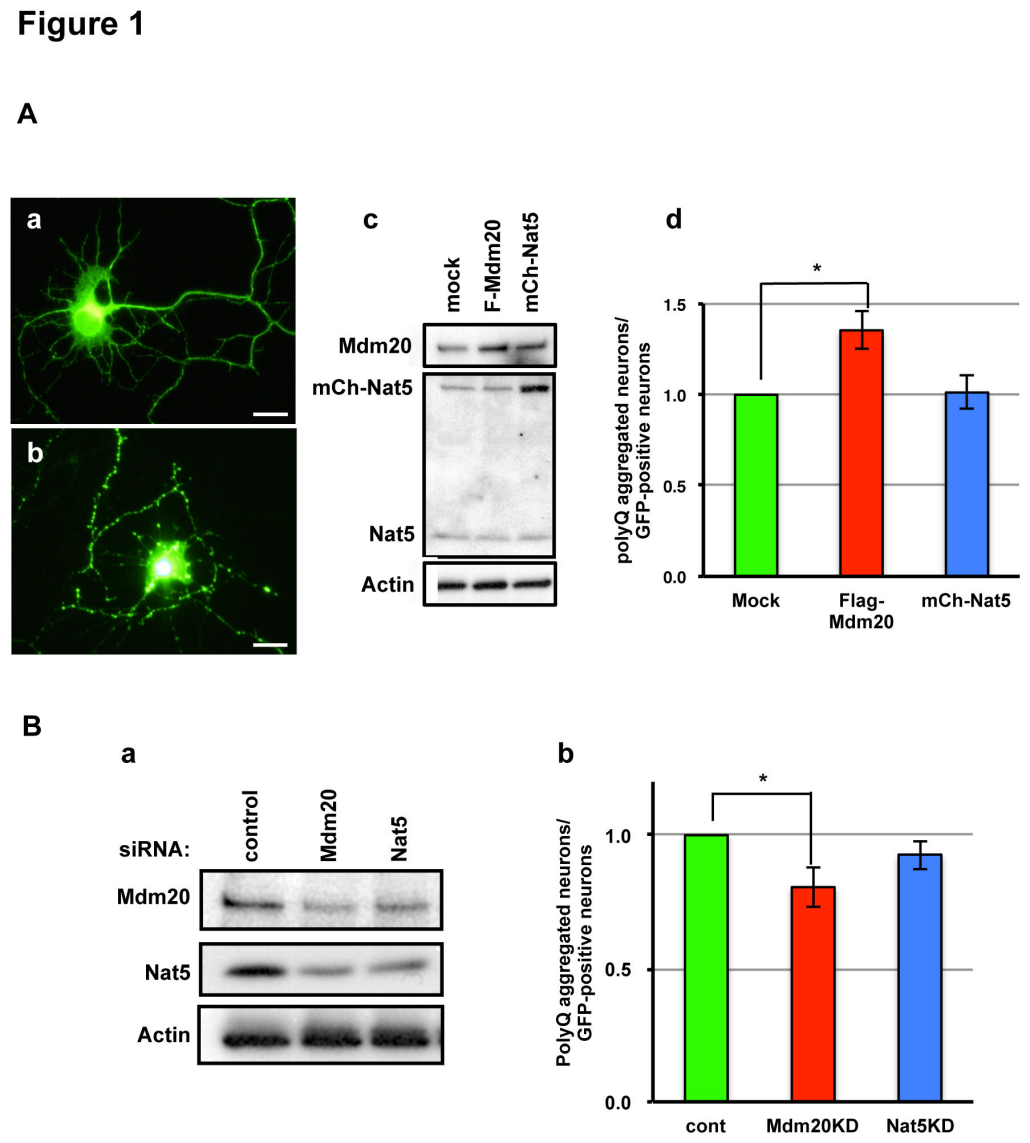
Mdm20 stimulates polyQ aggregate formation in hippocampal neurons. A. The number of polyQ aggregate-bearing neurons increase in Mdm20-OE rat primary cultured hippocampal neurons. Representative images of non- aggregated (a) and polyQ-aggregated neurons (b) after transfection of GFP-polyQ81 (Scale bar: 10 μm). Western blots of Mdm20, Nat5, and actin in transfected neurons (mock, Flag(F)-Mdm20 and mCherry(mCh)-Nat5) are shown in (c). The numbers of polyQ aggregate-positive neurons after transfection of GFP-polyQ81 with mock, F-Mdm20 or mCh-Nat5 were counted and compared with the number of GFP-expressing neurons. As in (d), the relative ratios were calculated with mock infection set at 1. The data represent the mean +/- S.D. (n=3) *P<0.0001. B. Mdm20-KD by siRNA reduces the number of polyQ-bearing cells. (a): A western blot showing the effectiveness of the siRNAs (control, Mdm20 and Nat5). (b): Evaluation of polyQ aggregates by neuron counting same as A-(d). *P<0.0001.

**Figure 2:**
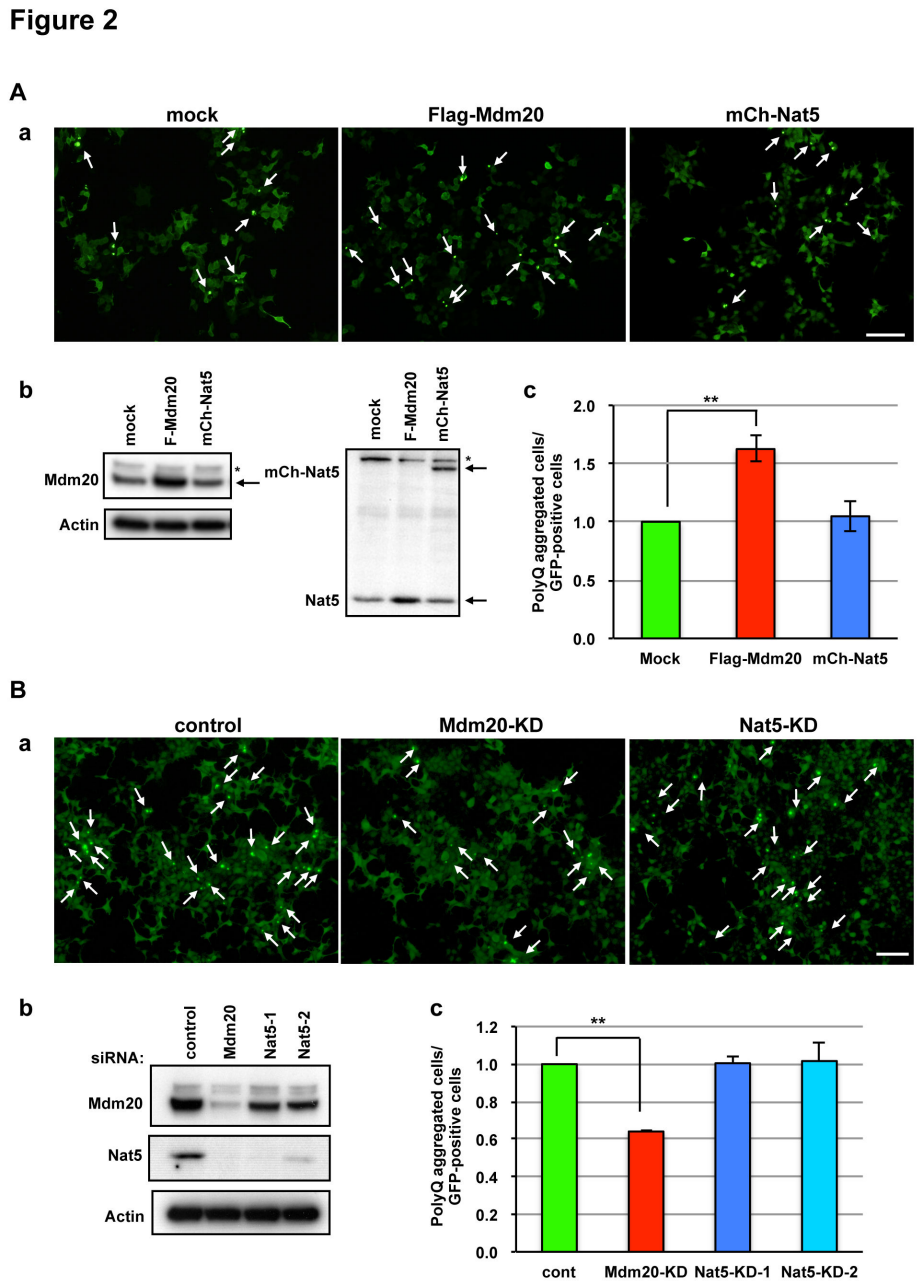
Mdm20 stimulates polyQ aggregate formation in HEK293 cells. A. The polyQ aggregate-bearing cells increase in Mdm20-OE HEK293 cells, but not in Nat5-OE cells. Upper panels (a) indicate the GFP-polyQ 81 transfected HEK293 cells with mock, Flag-Mdm20 or mCherry-Nat5. Arrows show the polyQ aggregate-positive cells. (Scale bar: 100 μm) Western blots of Mdm20, Nat5, and actin in transfected and non-transfected cells are shown in (b). The arrow indicates a correct Mdm20 or mCherry-Nat5 band. An asterisk denotes a cross-reacting band. As in (c), the polyQ aggregate-positive cells were counted and evaluated. The relative ratio of polyQ-bearing cells to GFP-positive cells was determined in Mdm20, Nat5, and mock transfected cells. **P<0.001. B. Mdm20-KD by siRNA reduces the number of polyQ-bearing cells. No difference was found between two sets of Nat5-KD cells and either Nat5-1 or Nat5-2 siRNAs. (a): GFP-polyQ aggregates formed HEK293 cells. (Scale bar: 100 μm) (b): A western blot showing the effectiveness of the siRNAs. Evaluation of polyQ aggregates by cell counting as in (c). **P<0.001.

To investigate protein aggregate formation and/or clearance in neurons, we used hippocampal neurons from E18 rat embryos. Flag-tagged Mdm20 or mCherry-tagged Nat5 and GFP-tagged polyQ81 constructs were co-transfected into primary cultured neurons at 5 DIV (days in vitro). At 48 hours post-transfection, both non-aggregated and polyQ aggregated cells were observed as GFP-expressing neurons (Figure 1A-a and b). Then, we counted the numbers of polyQ aggregates containing neurons and caliculated ther relative ratio of GFP-expressing neurons with non-polyQ aggregates. In Mdm20-over-expressing (OE) neurons, the polyQ-aggregated neurons increased (Figure 1A-d). In contrast, the Nat5–OE neurons had no effect on polyQ-aggregate formation. In addition, we examined these effects in Mdm20- or Nat5-siRNA transfected hippocampal neurons. Interestingly, although the amount of Nat5 was reduced in Mdm20-knockdown (KD) neurons, the amount of Mdm20 was slightly reduced in Nat5-KD neurons (Figure 1B-a). The numbers of polyQ aggregate formed neuron in Mdm20-KD neurons were markedly decreased in contrast to Mdm20-OE neurons (Figure 1B-b). On the other hand, polyQ aggregate formation was slightly suppressed in Nat5-KD neurons following the reduction of Mdm20 expression (see Figure 1 B-a and b). We confirmed these effects in HEK293 cells, which are well suited as a model for testing the effects of various constructs (Figure 2). Figures 2A-a and 2B-a showed the non-polyQ aggregated and polyQ aggregates formed GFP-expressing cells in NatB complex OE and KD HEK293 cells, respectively. Although the amount of Nat5 did not change in Mdm20-OE neurons (Figure 1A-c), they increased in Mdm20-OE HEK293 cells (Figure 2A-b). In contrast, although the amount of Nat5 was reduced in Mdm20-KD cells, the amount of Mdm20 was slightly reduced in Nat5-KD cells same as neuronal cells. (see Figures 1B-a and 2B-b). These data suggest that Mdm20 is an auxiliary factor for stabilization of Nat5. Similarly the cultured neurons, the number of polyQ aggregates-formed cells increased in Mdm20-OE HEK293 cells, and conversely reduced in Mdm20-KD cells. On the other hand, polyQ aggregate formation was not affected in Nat5-KD cells, similar to Nat5-OE cells (see Figure 2A and B). To confirm the reduction of polyQ aggregates formation in Mdm20-KD cells, we examined the amounts of polyQ aggregates using western blot analysis. In Mdm20-KD cells, the amounts of polyQ aggregates were reduced by about 60 percent as compared to control (see Figure S1). These data suggest that polyQ aggregate formation is affected by the amount of Mdm20.

**Figure 3:**
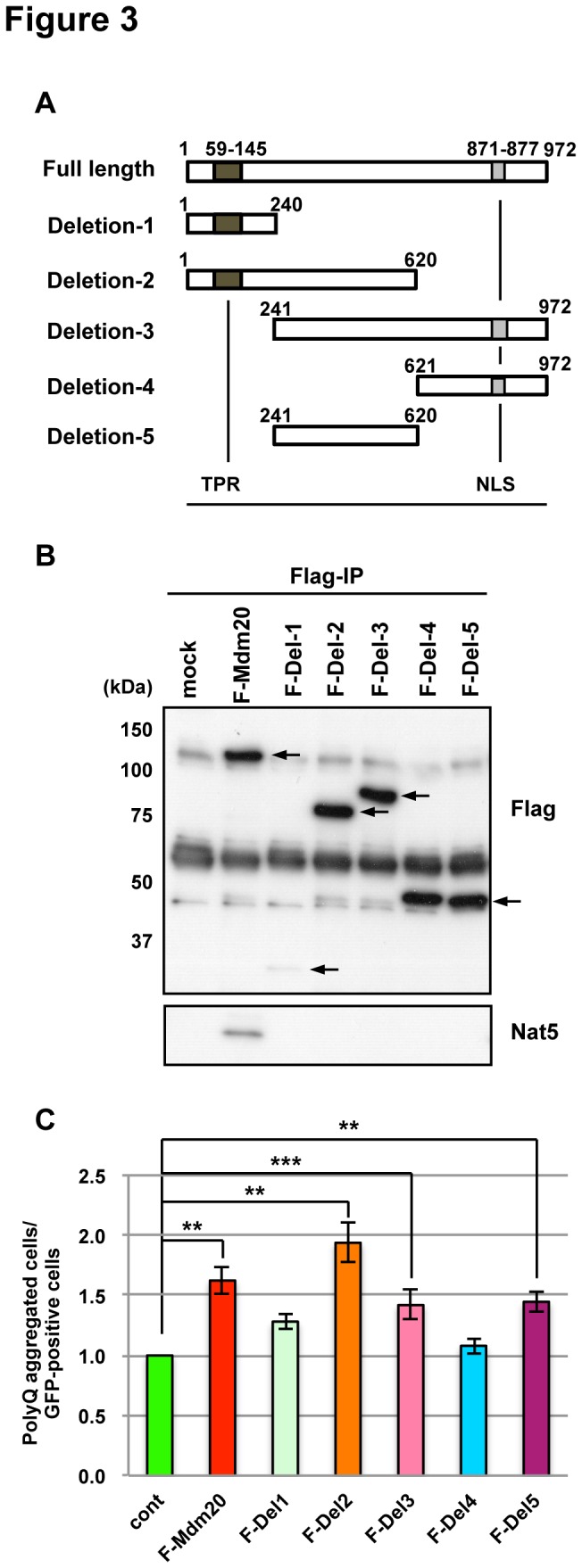
Delineation of the Mdm20 protein domains affecting polyQ aggregate formation: Independence from the Nat5-interaction. A. Schematic drawings of Mdm20 deletion constructs. The tri-peptide repeat (TRP) domain and a nuclear localization sequence (NLS) are indicated as black and gray squares, respectively. Amino acid residue numbers are given for each deletion. B. Western blots showing the expression efficiency of each Mdm20-deletion construct and the association with Nat5 in HEK293 cells. Forty-eight hours post-transfection, the cells were immunoprecipitated (IP) with an anti-Flag antibody and then immunoblotted with anti-Flag and anti-Nat5 antibodies. Note that only the full-length Mdm20 interacts with Nat5, and all the other partial deletion constructs fail to interact with Nat5. C. Comparison of the polyQ aggregate forming efficacy among the Mdm20-deletion constructs. The relative levels of polyQ-bearing cells are compared with the mock transfection without any Mdm20 constructs and only with the polyQ81 and GFP plasmids. The data represent the mean +/- S.D. (n=3) **P<0.001. ***P<0.01.

To identify the region of Mdm20 that interacts with Nat5, we made various Flag-tagged deletion mutants of Mdm20 (Figure 3A). Mdm20 has a TPR (tri-peptide repeat) domain at the N-terminal region. Using an immunoprecipitation assay with an anti-Flag antibody, endogenous Nat5 co-precipitated only with the full-length Mdm20 construct (Figure 3B). Additionally, we counted the numbers of polyQ aggregates formed cell in HEK293 cells transfected with each deletion mutant. Cells expressing deletion constructs 2, 3 and 5 which did not interact with Nat5, had increased polyQ aggregate formation, similarly the full-length Mdm20-expressing cells (Figure 3C). These data indicate that polyQ aggregate formation does not require the C-terminal region of Mdm20 or an interaction with Nat5. These results strongly suggest that Mdm20 regulates polyQ aggregate formation independently of its interaction with Nat5.

### The celluar localization of Mdm20 and colocalization with polyQ aggregates

**Figure 4:**
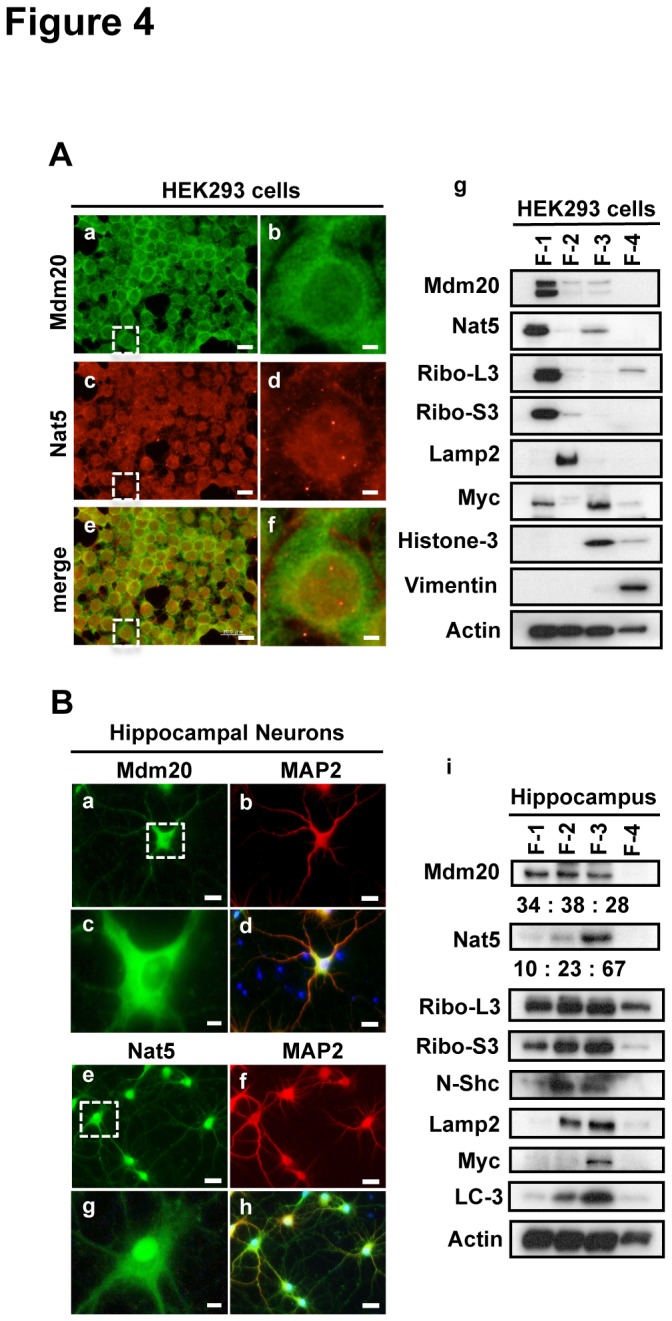
Immunocytochemical staining and cellular biochemical fractionation reveal distinct localizations of Mdm20 and Nat5. A. Distribution of NatB complex in HEK293 cells. Left: Immunocytochemistry of Mdm20 and Nat5 in HEK293 cells. After fixing HEK293 cells with paraformaldehyde, the cells were immunostained with anti-Mdm20 (green) and anti-Nat5 (red) antibodies. Shown are low magnification views (a,c,e) and partial-zoom views (b,d,f) of the area indicated (dot lined squares). (Scale bar: 20 μm (a, c, e), 3 μm (b, d, f)) Right: Cellular fractionation experiments in HEK293 cells. Western blots of Mdm20 and Nat5 together with other sub-cellular marker proteins are shown to reveal the efficiency of the cell fractionation process (g). F1, cytosolic fraction; F2, membranes and membrane organelles; F3, nuclear proteins; F4, components of cytoskeletal proteins. Marker proteins examined: Rpl3 and Rps3 (ribosomal proteins of large and small subunits, respectively), Lamp2 (marker of membrane fraction), Myc (marker of nuclear fraction), Histone3 (marker of nuclear chromatin fraction), vimentin (marker of cytoskeletal fraction), and actin (a general marker). B. Distribution of NatB complex in primary cultured rat hippocampal neurons. Left: Immunohistochemistry of Mdm20 and Nat5 in primary cultured rat hippocampal neurons at 7 DIV. Immunofluorescence analysis was performed for Mdm20 (a), Nat5 (e), and MAP2 (b,f) (a specific marker of neuronal dendrites). High-magnification images of Mdm20 and Nat5 staining (area as indicated with a dot-lined square) are shown (c,g). Merged images are also shown (d,h). (Scale bar: 20 μm (d, h), 5 μm (c, g)) (i): Cellular fractionation experiments of rat hippocampus at E18.5. Biochemical fractionations were performed as in A-(g) using brain regions from the hippocampi of embryos at E18.5. N-Shc was used as a neuron-specific marker protein. The relative intensities of the Mdm20 and Nat5 bands were quantified by densitometry and indicated below each blot.

We examined the sub-cellular distributions of Mdm20 and Nat5 in HEK293 cells. In an immunofluorescent experiment, Mdm20 broadly localized to the cytoplasmic region, while Nat5 localized mainly to the nucleus with additonal distribution in the cytoplasm at lower levels (Figure 4A-a-f). We further performed cellular fractionation. Approximately 80 % of Mdm20 was present in the cytoplasmic fraction, and only 10 % was present in the nuclear fraction. On the other hand, although the localization of Nat5 was primarily cytoplasmic, approximately 30 % of Nat5 was present in the nuclear fraction. Nat complexes are generally known to associate with poly- and mono-ribosomes [38]. Ribosomal protein L3 (Rpl3) and ribosomal protein S3 (Rps3) are composed of the 60S and 40S ribosomes, respectively. Yeast Nat3 and Mdm20 co-localize with mono- and poly-ribosomes, including Rpl3 and Rps3. In HEK293 cells, the fractionation patterns of Rpl3 and Rps3 were similar to that of Mdm20 (Figure 4A-g). Furthermore, we confirmed the cellular localization of Mdm20 and Nat5 in neurons. In rat primary cultured hippocampal neurons, Mdm20 mainly localized to the cell bodies and somewhat to the dendrites, as shown by the co-staining with MAP2 (Figure 4B-a-d). On the other hand, Nat5 was mainly localized to nuclear region (Figure 4B-e-h). These cellular fractionation experiments strongly indicate that the localization of Nat5 is mainly nuclear (approximately 70%) (Figure 4B-i). 

**Figure 5:**
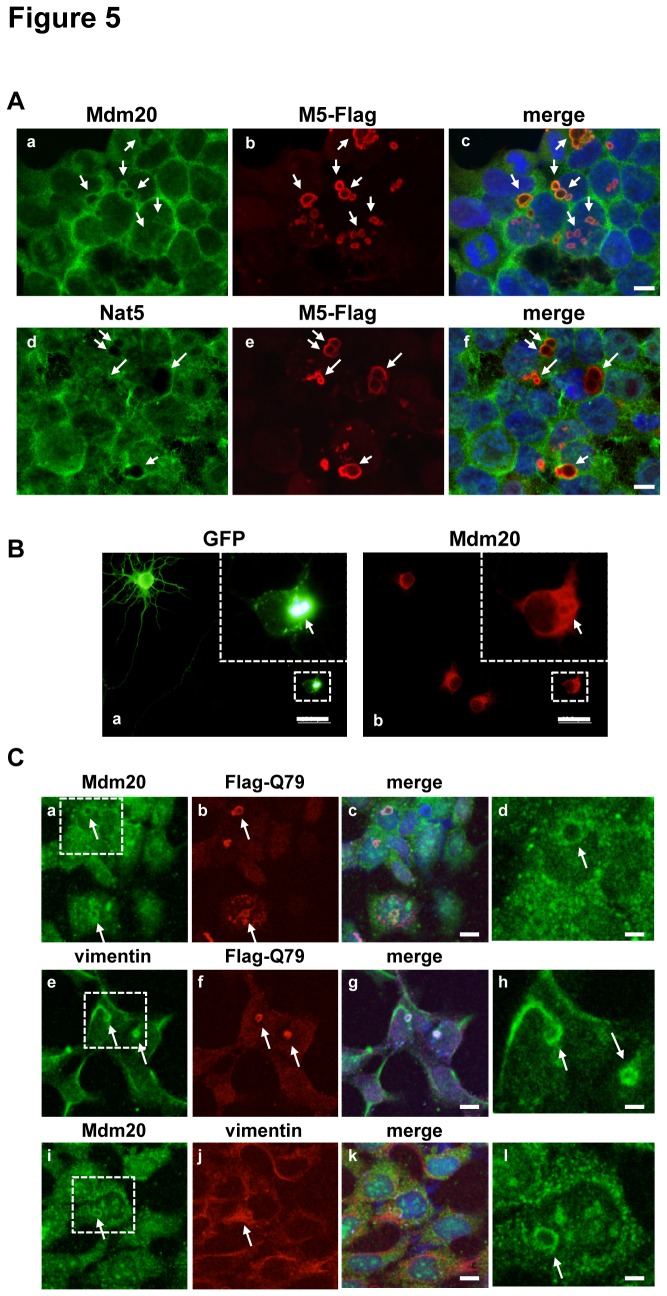
Mdm20 co-localizes with aggresome, wrapping the polyQ aggregates at the peri-nuclear region. A. Immunohistochemistry showing the cellular localization of Mdm20 (upper panels: a-c) and Nat5 (lower panels: d-f) following transfection with Flag-tagged Q79 containing plasmids into HEK293 cells. The cells were fixed, and the images were captured 48 hr post-transfection. The arrows indicate the polyQ aggregates at the perinuclear region. (Scale bar: 10 μm). B. Mdm20 co-localizes with polyQ aggregates, wrapping the aggregates in rat primary cultured hippocampal neurons. Immunohistchemistry shows the cellular localization of Mdm20 (b) following transfection with GFP-polyQ81 (a) into rat primary cultured hippocampal neurons. High-magnification images of GFP-polyQ81 and Mdm20 staining (area as indicated with a dot-lined square) are shown within each panels. The arrow indicates polyQ aggregates. (Scale bar: 20 μm). C. Mdm20 accumulates in areas surrounding polyQ aggregates. Immunostaining of Mdm20, vimentin and polyQ aggregates following transfection with a Flag-tagged polyQ79-containing plasmid is shown. The images in d,h,l are high-magnification images of areas in a,e,i. The arrows indicate polyQ aggregates at the periphery of nucleus. (Scale bar: 10 μm (c, g, k), 3 μm (d, h, l)).

We then tested whether the expression of Mdm20 correlates with the polyQ aggregates. To investigate the relationship between the cellular localization of the NatB complex and the polyQ aggregates, we transfected Flag-tagged Q79 (aggregate form) into HEK293 cells and performed immunofluorescence studies with anti-Flag and Mdm20 or Nat5 antibodies (Figure 5A). Aggregates of Flag-tagged Q79 localized to the peri-nuclear fraction as aggresomes. As expected, Mdm20 co-localized with Flag-tagged Q79 aggregates in HEK293 cells like a wrapping the aggregates, but Nat5 did not. In hippocampal neurons, Mdm20 colocalized with GFP-tagged Q81 aggregates same as HEK293 cells (Figure 5B). In addition, Mdm20 co-localized with vimentin, a marker protein of the aggresome, suggesting that Mdm20 indeed associates with polyQ aggregates (Figure 5C). These results further suggest that Mdm20 has biological functions that are independent of its interaction with Nat5 and effects the polyQ aggregate formation.

### Suppression of Mdm20 enhances autophagy

Based on our previous results, we wondered how Mdm20 regulates or participates in polyQ aggregate formation. As Mdm20 is a component of the NatB complex and participates in N-terminal acetylation during *de novo* protein synthesis, the overexpression or knockdown of Mdm20 may affect the rate or amount of total protein systhesis. To investigate whether the loss of Mdm20 affects protein synthesis, we performed *in vivo* labeling using [^35^S]-methionine (Met). However, the incorporation of [^35^S]-Met during protein synthesis did not differ between the control and Mdm20-KD HEK293 cells (data not shown), suggesting that Mdm20 does not regulate polyQ aggregation via protein synthesis or translational initiation. 

**Figure 6:**
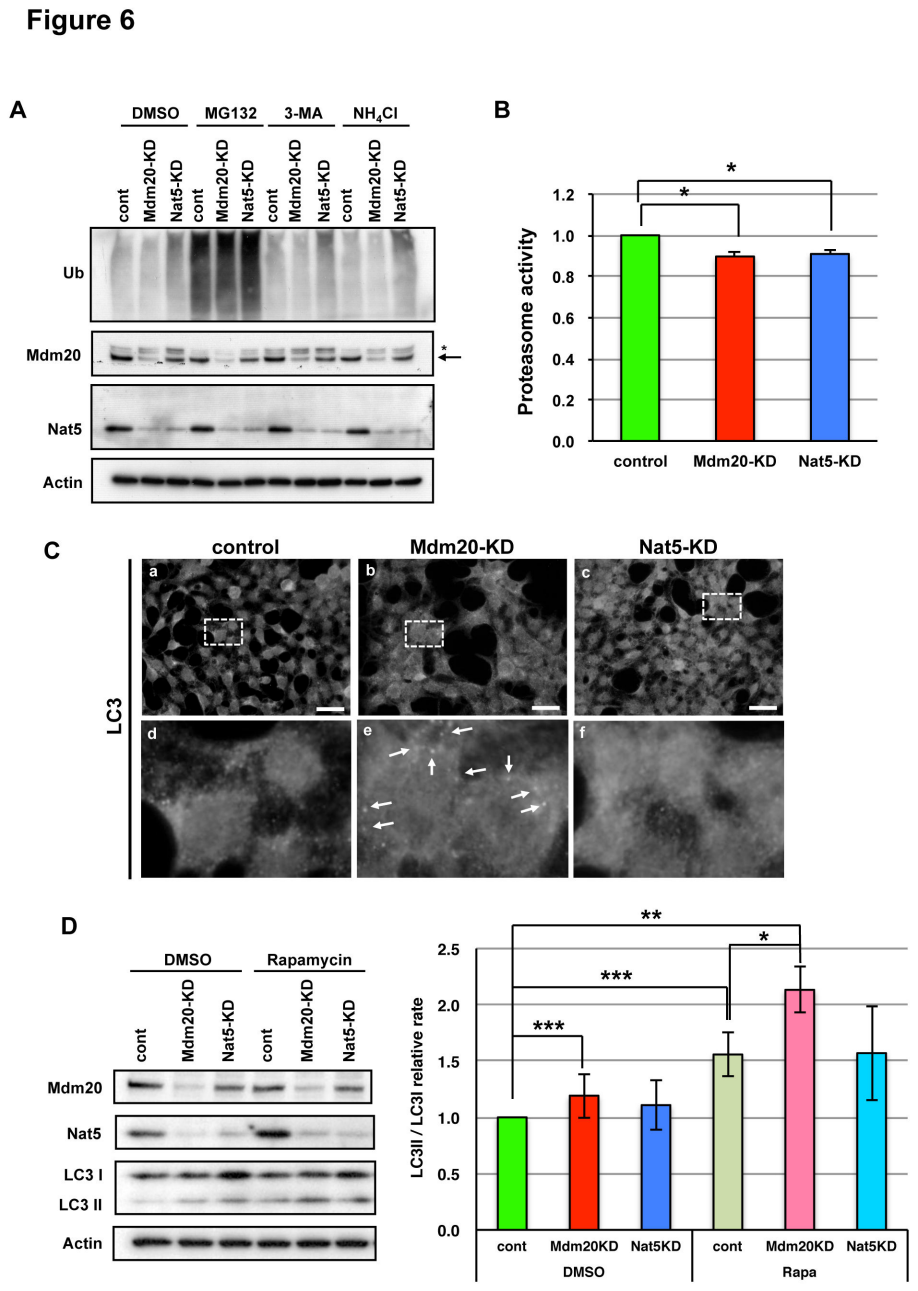
The effect of Mdm20 reduction on UPS and autphagy: Selective autophagy induction in Mdm20-KD cells. A. Western blots showing poly-ubiquitinated proteins (Ub) with or without transfection with siRNAs for Mdm20 and Nat5. To evaluate the effects of each siRNA, blots for Mdm20 and Nat5 were performed with β-actin controls. In the panel for Mdm20, the arrow indicates the bands at 120 kD corresponding to the expected size of Mdm20, and the asterisk denotes possible cross-reacting materials. B. Proteasome activity is unaffected in both Mdm20 and Nat5 knockdown in HEK293 cells. The activity of the proteasome was measured using a fluorescent-tagged polypeptide (Suc-LLVY-AMC) as a model substrate. Proteasome activities were calculated relative to the control cell extracts with control siRNA-transfected cells. The data represent the mean +/- S.D. (n=3) *P<0.01. C. Immunohistochemistry against LC3, a marker for autophagy, in Mdm20- and Nat5-KD cells. Panels d, e, f are high-magnification images of the area indicated in a, b, c. Note that punctate staining is apparent in the Mdm20-KD cells (e; allow). (Scale bar: 20 μm). D. Western blots for LC3-I and LC3-II to evaluate the induction of macroautophagy. DMSO, rapamycin (100 nM) are treated at 6h in control, Mdm20-KD, Nat5-KD HEK293cells. Note that the LC3-II bands are abundant in cells treated with rapamycin, in particular in Mdm20-KD cells. Actin was used as a loading control. The amounts of LC3II per LC3I were calculated relative to the control cell extracts with control siRNA-transfected cells. The data represent the mean +/- S.D. (n=5) *P<0.0001, **P<0.001, and ***P<0.01.

There are two main degradation systems that clear abnormal proteins like polyQ aggregates. One is a selective degradation system that functions through the ubiquitin-proteasome, and the other is a non-selective system that functions through a mechanism of autophagy. The proteasome selectively recognizes and degrades poly-ubiqutinated proteins using modified E1, E2 and E3 enzymes [39]. Under nutrient-deficient conditions, autophagy is induced by the insulin-like growth factor (IGF)-phosphatidyl inositol 3-phosphate kinase (PI3K)-Akt signaling pathway [40]. Autophagosome formation is regulated by various ATG genes, and the autophagosome eventually fuses with a lysosome, leading to the proteolytic degradation of the internal components of the autophagosome by the lysosomal enzymes [41]. To evaluate the role of Mdm20 in the protein degradation system, we used chemical inhibitors of protein degradation, i.e., MG132 (an inhibitor of the proteasome), 3-methyladenine (3-MA, an inhibitor of PI3K, which regulates autophagy induction), and ammonium chloride (an inhibitor of lysosome proteases). The amount of poly-ubiqutinated proteins was massively induced by MG132. This response did not change much under other conditions, although a slight upregulation was noted in the HEK293 cells with Nat5-KD, but it was not significant (Figure 6A). This result seems partially consistent with the observation in yeast that the Nat complexes acetylate the components of the proteasome, and NatA specifically regulates proteasome activity by N-acetylation [42,43]. Mdm20-KD using siRNA did not affect the levels of poly-ubiquitinated protein in HEK293 cells (Figure 6A). Additionally, while we examined the proteasome activity in Mdm20- and Nat5-KD cells, the activities were only slightly reduced in each condition (Figure 6B).

**Figure 7:**
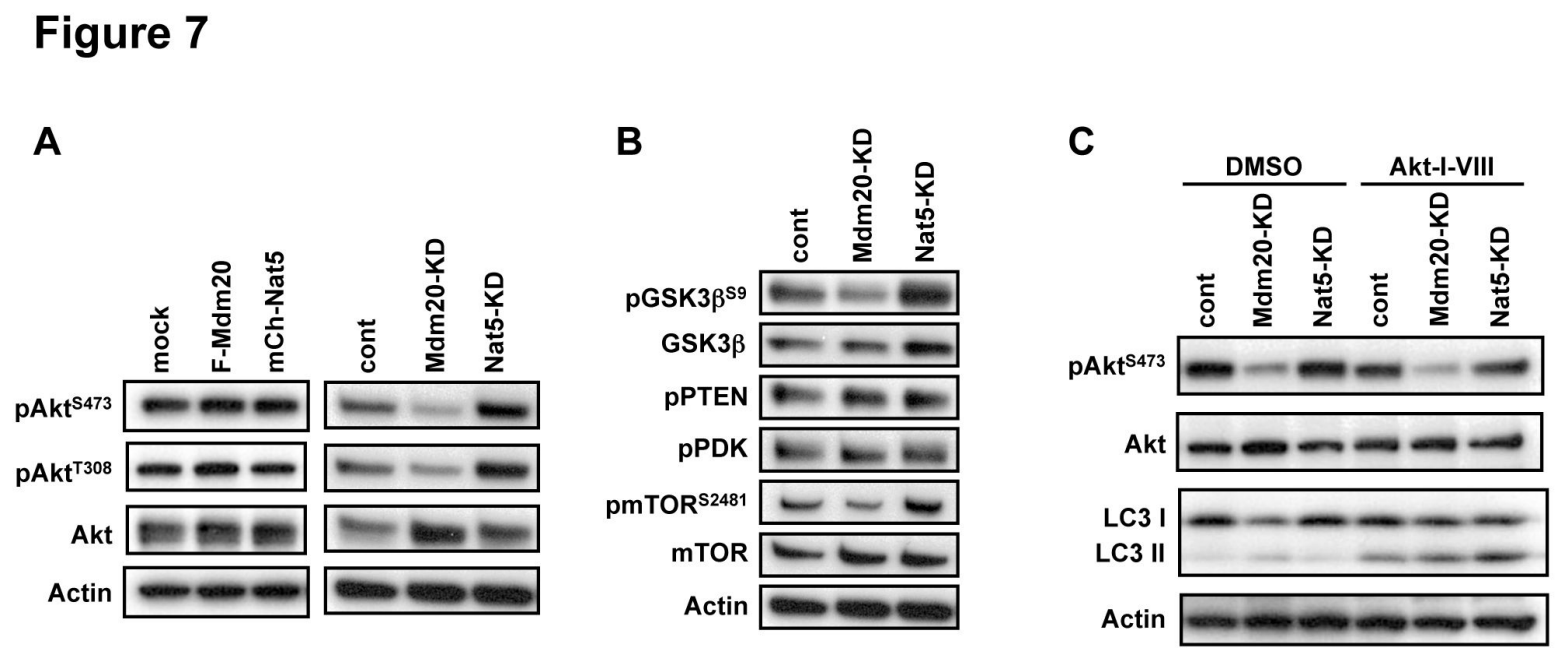
Mdm20 regulates the phosphorylation status of Akt. A. Western blots for Akt with a focus on phosphorylated Akt on Ser473 and Thr308. HEK293 cells were transfected either with mock, Flag-Mdm20 and mCherry-Nat5 or control, Mdm20 and Nat5 siRNA. At 48 hrs or 72hrs post-transfection, respectively, the cells were harvested, and cell lysates were prepared and processed for western blot analysis. The antibodies used are indicated. For details, see Materials and Methods. B. Western blots were used to determine the phosphorylation status of GSK3β, PTEN, PDK-1, mTOR, and PP1. The antibodies used were as indicated. Note that the phosphorylation of mTOR (Ser-2481) is consistently reduced in Mdm20-KD cells. C. Akt inhibition increases the levels of LC3II. Shown is western blots of HEK293 cellular extracts treated or untreated with siRNAs for Mdm20 or Nat5 in the absence (DMSO control) or presence of Akt inhibitor (Akt-I-VIII). Levels of Akt, and phospho-Akt (pAkt-Ser473), and LC3 levels are shown with the loading control of actin blot.

We then explored whether Mdm20 affects autophagy. A hallmark of autophagy induction is activation of LC3 or its cleavage to LC3-II [44]. To test the possible involvement of Mdm20 in the regulation of LC3 or autophagy, we performed immunofluorescent staining with an anti-LC3 antibody in HEK293 cells with or without the presence of siRNAs targeting Mdm20 or Nat5. In the naive condition, as shown in the control cells (Figure 6C a, d), LC3 staining was rather diffuse, but in the Mdm20-KD cells, punctate staining was evident (Fig. 6C b, e). This re-localization was not apparent in Nat5-KD cells (Figure 6C c, f). In a biochemical assay to examine the levels of LC3-I and LC3-II, we could hardly detect a slight induction of LC3-II in the Mdm20-KD cells, but not Nat5-KD cells. Moreover, this effect was enhanced in the condition in the presence of rapamycin (mammalian target of rapamycin (mTOR) inhibitor) and Akt-inhibitor-VIII (selectively inhibits Akt1/Akt2 activity), which can induce autophagy (Figures 6D and 7C). However, in Mdm20 or Nat5-OE cells, the amount of LC3II was not affected by these chemical treatments (data not shown). These results suggest that loss of Mdm20 induces and up-regulates the autophagy pathway, possibly leading to the stimulation of protein aggregate clearance. 

### Mdm20 affects phosphorylation levels of Akt, leading to protein aggregation control

Autophagy is regulated by the PI3K-Akt signaling pathway, which acts under the IGF signaling. Under nutrient-rich conditions or in adipocytes, Threonine-308 (Thr308) and Serine-473 (Ser473) of Akt are phosphorylated by activated PDK-1 and mTORC2/PDK2, respectively [45-48]. As the activated form of Akt phosphorylates tuberous sclerosis complex1 (TSC1) and 2, these complexes activate mTOR thorough Rheb, a member of the Ras family of small GTP-binding proteins [49,50]. Activated mTOR interacts with Raptor and GβL and suppresses ATG1, which is a major component of the initiation complex for autophagy induction [51,52]. While mTOR also interacts with Rictor and GβL, they phosphorylate Ser473 of Akt as mTORC2/PDK2. However, under conditions of starvation, as the phosphorylation level of Akt is markedly reduced, the activity of mTOR is suppressed, and autophagy subsequently is induced. 

To test whether Mdm20 regulates the PI3K-Akt-mTOR pathway leading to the induction of autophagy, we examined the phosphorylation level of Akt in Mdm20-KD HEK293 cells. To our surprise, although the amount of Akt was slightly increased, the phosphorylation level of Akt was markedly reduced in Mdm20-KD cells (Figure 7A). In contrast, the phosphorylation level of Akt was slightly increased in Mdm20-OE cells. The phosphorylation level of GSK3β, a well-known substrate of Akt, was also reduced in response to Akt activity. As expected, the phosphorylation level of mTOR, i.e., mTOR-Ser-2481, was also reduced similarly to that of GSK3β in Mdm20 KD cells (Figure 7B). However, all the aforementioned phosphorylation of these factors was unaffected in Nat5 KD cells (see lanes of Nat5-KD in each panel in Figure 7A and B). 

**Figure 8:**
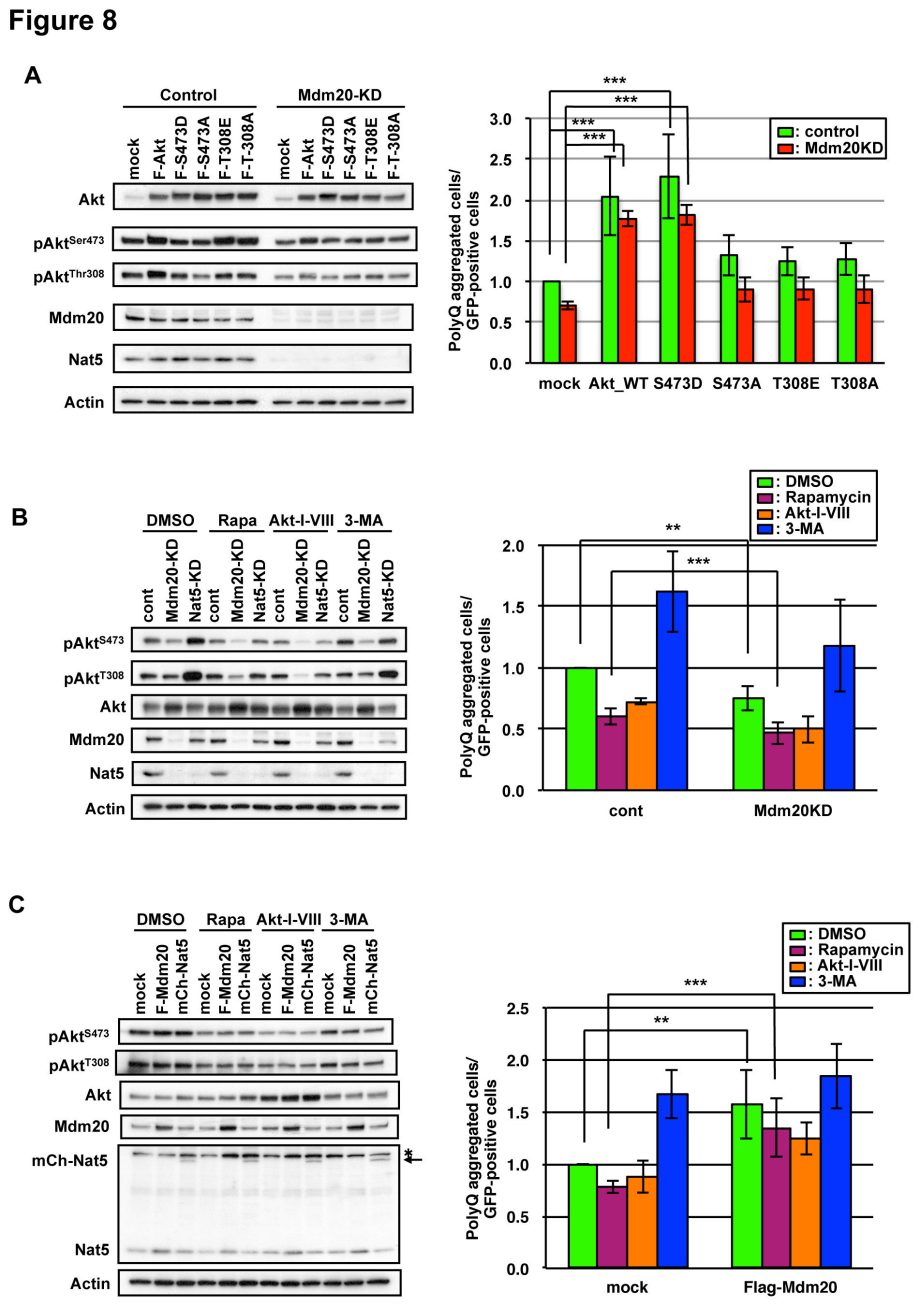
Mdm20 affects the polyQ aggregate formation by the regulation of pAkt^Ser473^ level. A. A phospho-mimic mutant of Akt (Akt-S473D) increases polyQ aggregate formation. Left: Shown are western blots for Akt and phospho-Akt (at Ser473 and Thr308). HEK293 cells were co-transfected with GFP-polyQ81 and Flag-tagged wild type Akt (F-Akt) or various phospho- and none-phospho-mimic Akt mutants as indicated. Right: The relative levels of polyQ aggregate formation were evaluated in each of the transfectants and compared with the mock-transfected control. Experiments were performed in naïve HEK293 cells (green bars) and Mdm20-KD cells (red bars). The numbers of polyQ-bearing cells among the GFP-positive cells were calculated and normalized to the level of mock transfection. The data represent the mean +/- S.D. (n=3) ***P<0.01. B. Mdm20-KD cells also reduce the phosphorylation level of Akt and polyQ aggregate formation. Left: The effects of Mdm20-KD on the phosphorylation level of Akt in the presense of various chemical treatment (DMSO, rapamycin (100 nM), Akt-inhibitor-VIII (5 μM) or 3MA (1 mM)). Right: The relative levels of polyQ aggregate formation were evaluated in each of the transfectants and compared with the mock-transfected control. The numbers of polyQ-bearing cells among the GFP-positive cells were calculated and normalized to the level of control transfection. The data represent the mean +/- S.D. (n=3) **P<0.001. ***P<0.01. C. Mdm20-OE cells also increase the phosphorylation level of Akt and polyQ aggregate formation. Left: The effects of Mdm20-OE on the phosphorylation level of Akt in the presense of various chemical treatment same as B. The arrow indicates a correct mCherry-Nat5 band. An asterisk denotes a cross-reacting band. Right: The relative levels of polyQ aggregate formation were evaluated in each of the transfectants and compared with the mock-transfected control. The numbers of polyQ-bearing cells among the GFP-positive cells were calculated and normalized to the level of mock transfection. The data represent the mean +/- S.D. (n=3) **P<0.001. ***P<0.01.

Conversely, the phosphorylation levels of Akt were influenced by protein phophatases. While Akt was dephosphorylated by PP1 [53], the amounts of phosphorylated PP1 (activated form) did not change among the cells examined (Figure 7B). On the other hand, the level of activated PDK1 was slightly increased in Mdm20-KD cells (Figure 7B). These results indicate that the activity of PI3K signaling, at least up to PDK1, was significantly activated by the loss of Mdm20 function. Akt is known to be phosphorylated first by mTORC2/PDK2 at Ser473 and further phosphorylated on Thr308 [54]. These results suggested that the expression level of Mdm20 regulates the phosphorylation status of Ser473 of Akt, and autophagy is subsequently induced through the activity of mTORC2. Consistent with this notion of autophagy induction, the amounts of LC3-II were apparently increased in Akt-inhibitor (Akt-I-VIII) treated cells, and this effect was slightly enhanced in Mdm20-KD cells, where level of Ser473 phopshorylation of Akt was significantly suppressed (Figure 7C). To further confirm the effect of the phosphorylation level of Ser473 of Akt on polyQ aggregate formation in Mdm20-KD cells, we constructed S473A and S473D mutants, which mimic the non-phosphorylated and phosphorylated forms of Akt, respectively. We co-transfected these mutants with GFP-tagged polyQ constructs. The phosphorylation levels of Thr308 of Akt in Akt-WT and Akt-S473D-OE cells were markedly increased, but this was not the case in Akt-S473A-OE cells (Figure 8A). In addition, the overexpression of Akt-WT and Akt-S473D increased polyQ aggregate formation in both control and Mdm20-KD cells (Figure 8A). These data indicate that regulation of the phosphorylation level of Ser473 by Mdm20 affects the progression of polyQ aggregation possibly through inhibiting autophagy induction.

Although rapamycin and Akt-inhibitor-VIII induce autophagy (Figures 6D and 7C)), they reduced the phosphorylation level of Akt (Figure 8B). In addition, these chemical treatments also suppressed the polyQ aggregate formation in mock or control cells (Figure 8B). Under these chemical treatment, the phosphorylation level of Akt was more reduced in Mdm20-KD cells and conversely more increased in Mdm20-OE cells. As corrrelation with the phosphorylation level of Akt, the number of polyQ aggregated cells were suppressed in chemical treated Mdm20-KD cells (Figure 8B and Supplememtal Figure S1). On the other hand, in Mdm20-OE cells, the number of polyQ aggregates formed cells was slightly decreased comapre to DMSO-treated Mdm20KD cells (Figure 8C). These data indicate that the pAkt level is well correlated with the polyQ aggregate formation and Mdm20 regulates the autophagy induction through the modulation of pAkt level. 

## Discussion

In the present study, we used polyQ aggregates as a model of neurodegenerative conditions to evaluate whether Mdm20 regulates protein clearance and/or aggregate formation in neurons as it does in budding yeast. We found that in neurons, Mdm20 promotes protein aggregate formation, and conversely, the loss of Mdm20 function leads to protein clearance by inducing autophagy through reducing the phosphorylation level of Akt-Ser473. Furthermore, this regulation by Mdm20 occured independently of Nat5; thus, our results suggest a novel function of Mdm20, which was previously believed to be merely an “auxiliary” subunit for protein N-acetylation.

There is significant evidence that Mdm20 interacts with Nat5 as a NatB complex, and furthermore, the complex is associated with ribosomes and/or polysomes. In fact, the amount of Nat5 increased in Mdm20-OE HEK293 cells and converserly decreased in Mdm20-KD-cells (see Figure 2A and B). These data suggest that Mdm20 is required for the stabilization of Nat5 and regulates the biological function of Nat5. However, in the present study based on mammalian cells, e.g., HEK293 and rat primary cultured hippocampal neurons, the fractionation pattern of Mdm20 correlated with ribosomal proteins in subcellular fractionation assay, but the fractionation pattern of Nat5 was distinct. This pattern is partially supported by the spatial distributions of the two proteins in HEK293 cells and neurons as evidenced by histological immunostaining. Thus, the localizations of Mdm20 and Nat5 are partly overlapped and partly distinct, and the degree also varies in each cell type. As aforementioned, Mdm20 seems to stabilize Nat5 protein as auxiliary subunit for the Nat complex. This could be true in the cytoplasm or around the polysomes; however, in the neuronal nucli Nat5 is quite abundant. In this case, possibilities remain either Nat5 is stablized by another factor in the nucleus, or Nat5 conformation varies in the nucleus to be stabilized yet recognized by the antibody.

More importantly, we clearly observed co-localization of Mdm20 with polyQ aggregates, which suggest that Mdm20 plays a role in aggresome formation along microtubule and/or actin-tropomyosin cytoskeletal cables as has been revealed in protein sorting and aging studies in yeast [34]. 

In budding yeast, the transport of protein aggregates requires actin-tropomyosin cables that are regulated by the NatB complex, of which Nat3 (yeast homolog of human Nat5) is the catalytic subunit for protein N-acetylation. Although Nat5 also retains N-acetyltransferase activity, we assume this activity may not be required for the regulation of polyQ aggregate formation. Because of, in mammalian cells, protein aggregate turnover may not rely on actin-tropomyosin cables unlike in yeast. Instead, aggregates, such as polyQ aggregates, are transported along microtubule networks toward aggresomes [55]. On the other hand, regarding the degradation pathways, abnormal proteins, including polyQ aggregates, are degraded by either UPS or autophagy. Nat family proteins acetylate various components of the 19S and 20S proteasome in budding yeast, and they regulate proteasome activity through N-acetylation [42,43]. However, in the present study, in mammalian cells, proteasome activity did not seem to be affected in the Mdm20- and Nat5-KD HEK293 cells. Instead of the UPS pathway, autophagy was apparently induced in Mdm20-KD cells, which is consistent with the punctate staining pattern of LC3 in autophagosome induction and the reduced level of Akt phosphorylation at Ser473 as a trigger of autophagy induction. Thus, we propose that Mdm20 could be a negative regulator of autophagy and/or protein clearance regardless of its association with Nat5. 

Although we demonstrated that Ser473 phosphorylation was markedly affected by Mdm20-KD, we assume that this is not a direct effect; in other words, there is some missing or unknown factor(s) that mediates the effect of Mdm20 on Akt modulation. Two sites of Akt, i.e., Thr308 and Ser473, are phosphorylated by PDK1 and mTORC2/PDK2, respectively, and those phosphorylation events occur in sequence; phosphorylation of Ser473 by mTORC2/PDK2 induces the subsequent phosphorylation of Thr308 by PDK1 [56]. Our results are consistent with this notion; in Akt-S473D-OE cells, the phosphorylation level of Thr308 was slightly increased, but not in Akt-S473A-OE cells (see Figure 8A). The primary site of phosphorylation in Akt at Ser473 seems to be sufficient for the progression of polyQ aggregation (see Figure 8A). We assume that Mdm20 positively regulates the activation of Akt affecting the phosphorylation of Ser473 presumably by mTORC2/PDK2.

While we suggest a new role of Mdm20, an auxiliary subunit NatB, in protein clearance via mTOR-Akt signaling, it should be noted that Ard1, a catalytic subunit of the NatA complex, also affects mTOR signaling by N-acetylation of TSC2 [57], which is a GAP (GTPase-activating protein) for Rheb (Ras homolog enriched in brain), a Ras family GTPase, and the active form of Rheb (Rheb-GTP) stimulates the phosphorylation of mTOR [58]. While TSC2 is known as a substrate of Akt, and phosphorylated TSC2 suppresses mTOR by inactivating Rheb from GTP- ot GDP-bound form [59]. These molecular interactions are complex, but the main target of Mdm20 may reside in these molecular networks. We examined whether Ard1 interacts with Mdm20 and regulate the PI3K-Akt-mTOR parthway instead of Nat5. However, Ard1 was not co-immunoprecipitated with Mdm20 in our assay (data not shown), suggesting that Mdm20 excerts it’s effects on mTOR in a distinct route from the Ard1-mediated regulation of the PI3K-Akt-mTOR pathway. 

Among a variety of kinases, Akt plays critical roles in the regulation of survival, inhibition of apoptosis, glucose metabolism, protein synthesis, and longevity [60]. In relation to its role in protein aggregation, huntingtin, a causal gene product of Huntington disease, is known to be phosphorylated by Akt under the influence of the IGF-PI3K pathway [61]. Akt thus functions in various critical steps in signal transduction for maintaining cellular homeostasis, leading to cell survival and/or longevity. As we showed here, Mdm20 can regulate the phosphorylation of Akt independently of its interaction with Nat5; however, we have not yet examined whether the N-acetylation activity is actually required for the regulation of Akt phosphorylation or not. In addition, PKA regulates polyQ aggregate formation through the phosphorylation of Heat shock factor 1 (HSF1), which is a transcription factor for HSP genes encoding molecular chaperones like HSP70 [62]. It is worth noting that a recent proteomic analysis of yeast NatB revealed that the N-acetylation activity of NatB affects the protein phosphorylation level [63] which may favor the hypothesis that Mdm20 may regulate the protein clearance by modulating the phosphorylation status including Akt and/or further upstream kinases. Although some deacetylases, for example Sirtuins and HDAC6, are known to be involved in protein aggregate control in neruonal aging [9,11-13,64], further investigation would be needed to elucidate the potential roles of acetylases, including protein N-acetylases such as NatB, in cellular control of proteostasis in long-lived cells such as neurons. At least, the reduction of acetylation level closely correlate with the protein homeostasis from our study and previous study of the biological function of Sirtuins and HDAC6.

Protein aggregation is a common feature of various age-related neurodegenerative diseases, including Alzheimer, Parkinson, and Huntington diseases. The hallmarks of these devastating brain disorders are protein aggregation and/or depositition of β-amyloid (plaque), α-synuclein (lewy body), or poly-glutamine (polyQ) [65]. It is currently unknown why such specifc deposits accumulate in specific neuronal subsets in patients with those diseases, but it should be awared that a protein’s life begins on a ribosome that is associated with the Nat complexs for protein N-acetylation. It would therefore be interestsing and important to understand whether components of the N-acetylation machinery are indeed involved in the regulation of protein turnover and/or homeostasis, i.e., proteostasis, in cells, particularly those with long lifespans such as neurons. Regardless of N-acetylation activity, the present finding of a novel role for Mdm20 would provide a new direction for the exploration of protein lifespan in long-living neurons in the brain. 

## Materials and Methods

### Animals

All the experimental procedures using animals were performed in accordance with institutional and national guidelines, and the experiments described here-in had been approved by the Institutional Animal Care and Use Committee of Nagasaki University (No. 1112200961). Efforts were made to minimize animal suffering and to reduce the number of animals used. The rats were housed in a room maintained at 24°C with an alternating 12:12 h light/dark cycle. Food and water were available ad libitum.

### Cell Culture and Transfection

HEK293 cells were maintained at 37°C in a humidified atmosphere of 5% CO_2_ and grown in Dulbecco’s modified Eagle’s medium (DMEM) containing 10% fetal bovine serum. Embryonic hippocampi were isolated from pregnant rats (Sprague Dawley) at E18.5, and the cells were dissociated and cultured using a standard protocol as described previously [66]. Transfection of HEK293 cells was carried out using Lipofectamine and Plus reagent (Invitrogen) at a density of 5 x 10^4^ cells or 1.5 x 10^5^ cells per well in 24- or 6-well plates, respectively. Rat primary cultures of hippocampal neurons (5 DIV) were transfected with expression plasmids using Lipofectamine LTX (Invitrogen) in cells plated at a density of 3 x 10^4^ cells per well in 24-well plates. Both cell lines were incubated for 48 hours after transfection. To perform siRNA transfection, 20 M solution of each oligonucleotide was added to the transfection cocktail (25 nM, final conc.) and transfected into hippocampal neurons (3 DIV) or HEK293 cells using Lipofectamine2000 (Invitrogen), and the cells were incubated for 72 hours. 

### Plasmids and siRNA Oligonucleotides

Flag-tagged Mdm20 was a gift from Dr. Hotokezaka. Deletion constructs of Mdm20 were generated by a conventional method using PCR-primers (Table S1) and subcloned into the pCMV-Flag vector at the EcoRI and XhoI sites. Human Nat5 cDNA was obtained from Kazusa DNA Res. Inst. and was subcloned into a mCherry vector (Promega). GFP-tagged polyQ45 and Q81 as well as Flag-tagged polyQ35 and Q79 have been described previously [67]. The Akt plasmid was a gift from Dr. Kikkawa [68], and the Akt-S473A and S473D expression plasmids were constructed by site-directed mutagenesis (Stratagene). siRNA oligonucleotides targeting Mdm20 and Nat5 were chemically synthesized (Integrated DNA Technologies/IDT), and the sequences are shown in Table S2. The control siRNA was purchased from IDT.

### Antibodies and Chemicals

A rabbit polyclonal antibody was generated against C-terminal peptides of human Mdm20 protein, which is conserved between human and mouse, fused to the GST protein. The details have been described previously [33]. The anti-Nat5 (human homologue of yeast Nat3) antibody was purchased from Santa Cruz. The other antibodies used in this study were purchased as follows: anti-Flag, anti-β-actin and anti-MAP2 were purchased from Sigma; anti-ribosomal protein L3, anti-Myc, anti-vimentin and anti-PP1 antibody were purchased from Santa Cruz; anti-phospho-Akt (Ser473 and Thr308), anti-Akt, anti-GSK3β, anti-phospho-GSK3β, anti-mTOR, anti-phospho-mTOR (Ser2481), anti-phospho-PDK (Ser241) were purchased from Cell Signaling Technology; anti-LC3 was purchased from Medical and Biological Laboratories (MBL); anti-ubiquitin was purchased from Millipore; anti-GFP was purchased from Nacalai Tesque; MG132 and 3-methyladenine were purchased from Sigma, ammonium chloride was from Nacalai Tesque, rapamycin was from Cell Signaling Technology and Akt inhibitor VIII was from Merck.

### Immunoblotting

After the chemical treatments, the cells were washed with PBS and lysed with RIPA buffer containing 50 mM Tris-Cl (pH7.5), 150 mM NaCl, 5 mM EDTA, 1 % NP-40, 0.1 % SDS, 1 mM DTT, a phosphatase inhibitor mix (Nacalai Tesque) and a protease inhibitor cocktail (Nacalai Tesque) on ice. The cell lysates were subjected to immunoblotting with the indicated antibodies, and the immune complexes were detected with a chemiluminescence reagent (GE Healthcare).

### Immunofluorescence Analysis

HEK293 cells or rat primary cultured hippocampal neurons were fixed with 4 % paraformaldehyde and permeabilized with PBS and 0.2% Triton-X100. The cells were blocked with 1 % albumin in PBS and incubated with primary antibodies. Subsequently, cells were treated with the appropriate secondary antibody as follows: Alexa 488 donkey anti-rabbit IgG, Alexa 488 donkey anti-goat IgG, Alexa 488 donkey anti-mouse IgG, Alexa 594 donkey anti-rabbit IgG, Alexa 594 donkey anti-mouse IgG, HRP-conjugated anti-mouse IgG (Molecular Probes). The DNA was counterstained with DAPI (Dojindo). Images were captured using an Axioskop2 plus (Zeiss) fluorescent microscope with Axiovision software.

### Quantification of PolyQ Aggregates

GFP-polyQ81 alone or with the expression plasmids described above were transfected into either HEK293 cells or rat primary hippocampal neurons on 24 well plates. After the culture for 48 or 72 hours, GFP-positive cells with or without aggregates were counted, separately. We then calculated the ratio of the number of cells with GFP-polyQ aggregates versus that of all GFP-positive cells. Note that, when GFP-polyQ81 alone was transfected, 30-50 % of the GFP-expressing cells formed polyQ aggregates. Typically, one aggregate in the perinuclear region of the cells as shown in Figure 1A-b.

### Subcellular Fractionation

The ProteoExtract Subcellular Proteome Extraction Kit (Calbiochem) was used for subcellular fractionation. Briefly, HEK293 cells were plated at a density of 1 x 10^6^ cells per 9-cm dish and incubated for 16 hours before extraction. Embryonic hippocampi were isolated from pregnant rats (Sprague Dawley) at E18.5. Extracts of these samples were prepared using the kit as outlined in the supplier’s protocol. Equal volumes of each fraction were subjected to SDS-PAGE and blotted onto PVDF membranes.

### Proteasomal Assay

Approximately 1 x 10^5^ HEK293 cells were transfected with siRNA oligonucleotides and incubated for 72 hours. The cells were lysed in CHAPS buffer containing 50 mM Tris-HCl (pH 7.5), 100 mM NaCl, 0.2% CHAPS, 5 mM EDTA, 1 mM EGTA, 3 mM NaN_3_, and a protease inhibitor cocktail (Nacalai Tesque), and the cell extracts were clarified by centrifugation. For measurement of the chymotrypsin-like peptidase activity of the proteasome, Succinil-Leu-Leu-Val-Tyr-7 amino-4-methylcoumarin (Suc-LLVY-AMC; PEPTIDE Inst.) was prepared from a 10-mM stock solution in DMSO to yield a final concentration of 50 M. The substrates were diluted in 50 mM Tris-HCl (pH 7.5), 100 mM NaCl, 5 mM EDTA, 1 mM EGTA, 3 mM NaN_3_, and 2 mM DTT. A total of 80 μl of cell extract was incubated with 100 μl of the substrate solution for 15 min at 37°C. The fluorescence of the released AMC (7-amino-4-methylcoumarin) was measured with an excitation wavelength of 355 nm and an emission wavelength of 460 nm (FLUOstar OPTIMA; BMG LABTECH).

### Statistical Analysis

Statistical analyses were performed using Microsoft Exell. Comparisons were made using the t-test. The data were presented as the mean +/- standard deviation (S.D.). *P* values of less than 0.01 were considered as statistically significant. 

## Supporting Information

Figure S1 **High-molecular weight polyQ-aggregates are reduced in Mdm20-KD cells as evidence by biochemical analysis.** HEK293 cell extracts, treated or untreated with rapamycin (Rapa) in the presence or absence (control) of siRNAs for Mdm20 or Nat5, were fractionated to soluble (a) and precipitate (b) fractions, and further subjected to western blots using GFP antibody. Soluble low molecular weight GFP-polyQ81 migrates around 40 kD (arrow), whereas high molecular weight GFP-polyQ aggregates stay on the top of the gel (asterisk). Note that the amounts of high molecular weight polyQ aggregate were reduced in the presence of Mdm20 siRNA. Similarly, in the presence of rapamycin which induces autophagy, Mdm20-KD further reduced the polyQ aggregates formation. Relative intensities of the high molecular weight bands (asterisk) are shown at the bottom of the gel profile.
(TIF)

Table S1 **We used the primer sets to cunstruct the deletion mutants and showed below.** Deletion-1: Mdm20-5term/Mdm20-Del3 (PCR product: 720 bp). Deletion-2: Mdm20-5term/Mdm20-Del4(PCR product: 1860 bp). Deletion-3: Mdm20-Del1/Mdm20-3term (PCR product: 2193 bp). Deletion-4: Mdm20-Del2/Mdm20-3term (PCR product: 1053 bp). Deletion-5: Mdm20-Del1/Mdm20-Del4 (PCR product: 1137 bp). These PCR products were digested by EcoRI and XhoI and subcloned into pCMV-Flag vector.
(DOC)

Table S2 **We used these sequences for siRNA oligonucletides.** The numbers indicate the coding regions, which use for synthesize the siRNA oligonucleotides.
(DOC)
