# Supplementary figures and images for: Mdm20 Stimulates PolyQ Aggregation via Inhibiting Autophagy Through Akt-Ser473 Phosphorylation
Source: PLoS One. 2013 Dec 16;8(12):e82523. doi: 10.1371/journal.pone.0082523 (PMC3865000; doi:10.1371/journal.pone.0082523)

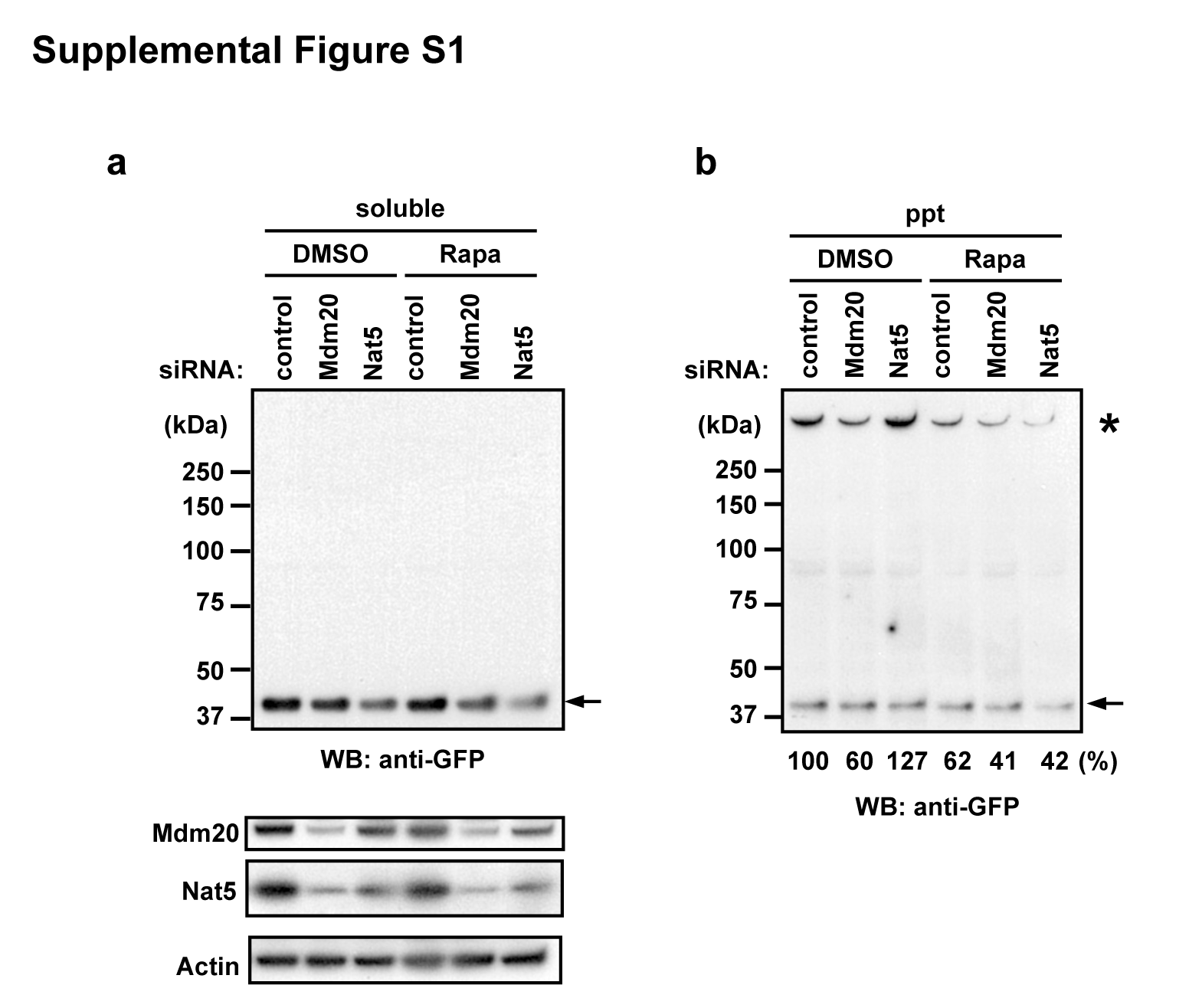

Supplement: Figure S1 — High-molecular weight polyQ-aggregates are reduced in Mdm20-KD cells as evidence by biochemical analysis. HEK293 cell extracts, treated or untreated with rapamycin (Rapa) in the presence or absence (control) of siRNAs for Mdm20 or Nat5, were fractionated to soluble (a) and precipitate (b) fractions, and further subjected to western blots using GFP antibody. Soluble low molecular weight GFP-polyQ81 migrates around 40 kD (arrow), whereas high molecular weight GFP-polyQ aggregates stay on the top of the gel (asterisk). Note that the amounts of high molecular weight polyQ aggregate were reduced in the presence of Mdm20 siRNA. Similarly, in the presence of rapamycin which induces autophagy, Mdm20-KD further reduced the polyQ aggregates formation. Relative intensities of the high molecular weight bands (asterisk) are shown at the bottom of the gel profile. (TIF) [file pone.0082523.s001.tif]
