# Supplementary material for: Mdm20 Stimulates PolyQ Aggregation via Inhibiting Autophagy Through Akt-Ser473 Phosphorylation
Source: PLoS One. 2013 Dec 16;8(12):e82523. doi: 10.1371/journal.pone.0082523 (PMC3865000; doi:10.1371/journal.pone.0082523)
Supplement: Table S1 — We used the primer sets to cunstruct the deletion mutants and showed below. Deletion-1: Mdm20-5term/Mdm20-Del3 (PCR product: 720 bp). Deletion-2: Mdm20-5term/Mdm20-Del4(PCR product: 1860 bp). Deletion-3: Mdm20-Del1/Mdm20-3term (PCR product: 2193 bp). Deletion-4: Mdm20-Del2/Mdm20-3term (PCR product: 1053 bp). Deletion-5: Mdm20-Del1/Mdm20-Del4 (PCR product: 1137 bp). These PCR products were digested by EcoRI and XhoI and subcloned into pCMV-Flag vector. (DOC) [file pone.0082523.s002.doc]

### Table S1

| **Mdm20-5term** | CGGAATTC**GGCACGAGCGCTTGTCATCA** |  |  |
| --- | --- | --- | --- |
| **Mdm20-Del1** | CGGAATTC**GATGAAGCTGAGCAGGTGGCCAGA** |  |  |
| **Mdm20-Del2** | CGGAATTC**GATGCTTCTACTTGAAGCAAATAT** |  |  |
| **Mdm20-Del3** | CCGCTCGAG**TCACTTGTACATAGCCATGCATT** |  |  |
| **Mdm20-Del4** | CCGCTCGAG**TCAGTCTAACAGCATCCGTTCAG** |  |  |
| **Mdm20-3term** | CGGAATTC**CTAGATATCTCGAGTGCGGCC** |  |  |
