# Supplementary material for: Mdm20 Stimulates PolyQ Aggregation via Inhibiting Autophagy Through Akt-Ser473 Phosphorylation
Source: PLoS One. 2013 Dec 16;8(12):e82523. doi: 10.1371/journal.pone.0082523 (PMC3865000; doi:10.1371/journal.pone.0082523)
Supplement: Table S2 — We used these sequences for siRNA oligonucletides. The numbers indicate the coding regions, which use for synthesize the siRNA oligonucleotides. (DOC) [file pone.0082523.s003.doc]

### Table S2

| **Mdm20-KD1 (1774-1792)** | AGCGAUAAACUCUGGGAUC |  |  |
| --- | --- | --- | --- |
| **Mdm20-KD2 (329-347)** | UGAUACUCCUCACUAUUGG |  |  |
| **Nat5-KD1 (67-85)** | CUUACAGAAACUUAUGGGA |  |  |
| **Nat5-KD2 (45-63)** | CAACAACAUUAACUUGGAU |  |  |
